# Hemispheric consistency in language production, comprehension, and reading in typically and atypically lateralized left-handers: Implications for reading performance

**DOI:** 10.1162/IMAG.a.1240

**Published:** 2026-05-26

**Authors:** Esteban Villar-Rodríguez, María Baena-Pérez, Cristina Cano-Melle, Adrian Nicolae, Lidón Marin-Marin, César Avila

**Affiliations:** Neuropsychology and Functional Neuroimaging, Universitat Jaume I, Castelllón de la Plana, Spain; Basque Center on Cognition, Brain and Language, Donostia-San Sebastián, Spain

**Keywords:** language lateralization, hemispheric specialization, speech production, auditory comprehension, overt reading, left-handedness

## Abstract

Language is a complex cerebral function comprising several processing components, such as speech production, auditory comprehension, and reading. While speech production is functionally specialized in the left hemisphere for most of the population, recent findings suggest that receptive components may follow an independent pattern of hemispheric lateralization. In this study, we used fMRI to analyze a sample of 69 left-handed participants, preselected to include a higher-than-usual proportion of people with atypical hemispheric lateralization. We assessed language functional lateralization across three components: speech production (Broca’s area), auditory comprehension (Wernicke’s area), and overt reading (visual word form area). Using the Laterality Index—a measure of functional hemispheric lateralization—we found a remarkably strong concordance between speech production and auditory comprehension (ρ = .653), with reading emerging as the least concordant of the three language components (ρ = .382 and ρ = .307, with production and comprehension, respectively). Based on these results, we categorized participants as either consistently lateralized (all components lateralized to the same hemisphere; n = 44) or inconsistently lateralized (one component diverging from the others; n = 25). Using this classification, we found that hemispheric inconsistency was predominantly characterized by an overall rightward dominance of language (two components lateralized to the right and one to the left), with reading being the most common diverging component. As a result, a left-lateralized function diverging from an overall rightward dominance was more common than the reverse pattern. Crucially, we also observed that hemispheric inconsistency—both as a categorical and continuous measure—was associated with slightly slower reading speed, particularly when the inconsistency occurred between speech production and reading components. In conclusion, our findings challenge previous accounts by showing that speech production and auditory comprehension lateralize quite uniformly, and that exceptions to this—inconsistent patterns mostly involving a divergent lateralization of reading—are associated with a slight reduction in reading speed.

## 1 Introduction

Speech production is generally lateralized to the left hemisphere of the brain (Broca, 1861; Tremblay & Dick, 2016). However, certain individuals exhibit this linguistic function predominantly in the right hemisphere or with an equal contribution from both halves of the brain (Ocklenburg & Güntürkün, 2024). This unusual phenomenon—known as atypical lateralization of language—occurs in approximately 4–6% of right-handers and 22–27% of left-handers when assessed using a functional magnetic resonance imaging (fMRI) language production task (Mazoyer et al., 2014; Pujol et al., 1999). Still, language is a complex function composed of multiple processing components, such as articulation and comprehension, which rely on different neural circuits (for reviews, see: Fedorenko et al., 2024; Price, 2012). To what extent, then, is hemispheric lateralization uniform across these different components, both in typical (left) and atypical (right or ambilateral) lateralization? Furthermore, are different hemispheric organizations associated with differences in behavioral outcomes, such as linguistic performance?

The dual-stream processing model of Hickok and Poeppel (2007) provides a framework for understanding how the cerebral lateralization of linguistic functions arises. According to this model, language relies on two major pathways linking temporal auditory regions with frontal and parietal cortices. The dorsal stream supports the transformation of speech sounds into motor plans and is, therefore, involved in speech production, repetition, and phonological operations. This pathway shows the strongest left-hemispheric dominance. The ventral stream, in turn, maps sounds onto meanings and is largely bilateral in the temporal cortex, although the left hemisphere plays a greater role in lexico-semantic processing and the right hemisphere contributes to prosody and pragmatic interpretation. In this framework, lateralization is not uniform across the language network. Early auditory processing is bilateral, but left-hemispheric dominance emerges as information becomes more abstract and phonologically encoded. Dorsal-stream functions are the most consistently left-lateralized, whereas many temporal functions—especially those within the ventral stream—are more symmetrically distributed across hemispheres.

Studies on hemispheric language lateralization have presented results that are quite consistent with this dual-stream model. Hemispheric lateralization during speech production has been extensively studied in the inferior frontal gyrus, both in its typical (leftward) and atypical (rightward) expressions (Mazoyer et al., 2014; Pujol et al., 1999; Springer et al., 1999; Szaflarski et al., 2002). However, lateralization during linguistic functions other than speech production has received less attention, particularly in healthy populations (Bradshaw, Thompson, et al., 2017). The overall pattern for comprehension and reading tasks, which are functionally localized in more posterior brain areas, shows lower lateralization values (i.e., more bilateral) in right-handers, left-handers, and patients with lesions (Parker et al., 2022; Price, 2010; Stroobant et al., 2009; Van der Haegen et al., 2012; Zaca et al., 2012). This pattern, however, may depend on other factors such as the task baseline or the studied brain regions (Bradshaw, Bishop, et al., 2017; Price, 2010). For this reason, we consider it relevant not only to compare the lateralization of different language tasks across studies, but also to examine the phenomenon of co-lateralization in the same sample while incorporating both typical and atypical forms of language lateralization. In an initial study, Van der Haegen et al. (2012) used fMRI to investigate co-lateralization between a lexical decision reading task and a word generation task in participants selected for their high probability of presenting atypical lateralization based on a tachistoscope hemifield test. Their results showed a lower lateralization in the ventral occipitotemporal region during reading than in the inferior frontal gyrus during production, but still, both lateralizations were highly correlated (r = .59). Gerrits et al. (2019) obtained very similar results using a word recognition 1-back task (ρ = .65). Similarly, Häberling et al. (2016) found not only a high level of co-lateralization between a word generation task (measured in the inferior frontal gyrus) and a synonym judgment test (measured in the medial temporal gyrus), but also very similar lateralization magnitudes. Finally, Parker et al. (2022) used functional transcranial Doppler (fTCD; a non-invasive ultrasound technique that compares the changes in arterial blood flow velocity between both hemispheres) and a larger sample size (230 participants) to study six different language tasks in left- and right-handers. By applying structural equation modeling on the lateralization indices (LIs, the most widely used numerical measure of functional lateralization), they showed that a two-factor solution explained a larger percentage of variance than a single-factor one. Notably, these two factors resembled—but did not clearly correspond to—the two components proposed by the dual-stream model.

Beyond the averaged group-level data, subject-wise analyses have revealed a percentage of cases with clear inconsistencies between their LIs. This pattern has been found in right-handers (Gurunandan et al., 2020) and in left-handers (Gerrits et al., 2019; Labache et al., 2020; Tzourio-Mazoyer et al., 2004) using production, comprehension, and reading tasks. Labache et al. (2020) noted not only the existence of hemispheric inconsistencies in language—two components lateralized in different hemispheres—but also that this phenomenon was significantly more prevalent among the atypically lateralized individuals (40% incidence in a sample of 30 participants) in comparison with the typically lateralized group (4% incidence in a sample of 257 participants). This is in line with a previous PET study by Tzourio-Mazoyer et al. (2004), who described hemispheric inconsistencies between generation and comprehension in 1 individual out of 6 atypical participants, but in no individual out of 14 typicals. In patients, chronic epilepsy has been associated with hemispheric dissociations of production and comprehension (Lee et al., 2008). So, current evidence indicates that the different components of language processing—and thus the different regions of the language network—can lateralize independently, both in magnitude and direction.

Another, separate question is whether these differential patterns between posterior and anterior regions have consequences for the performance of language tasks. Asymmetric cerebral processing has been proposed to entail certain cognitive advantages, mostly related to more efficient parallel processing and faster processing speed (Ringo et al., 1994; Rogers, 2000; Vallortigara & Rogers, 2020). Earliest theories of language lateralization also introduced the idea that different asymmetry profiles might provide the individual with different sets of advantages and disadvantages (Geschwind & Galaburda, 1985; Mcnamara et al., 1994). As a result, several studies have explored the potential impact of hemispheric language dominance on linguistic performance. Overall, evidence on this relationship remains inconclusive, with studies reporting both positive and negative results. It is important to consider that sample sizes and statistical power may contribute to these inconsistencies, as atypically lateralized individuals are relatively rare, leading to small atypical groups in most studies (for a review, see Quin-Conroy et al., 2024). In the following section, we discuss the studies most relevant to the current investigation.

Knecht et al. (2001) used fTCD during a phonological fluency task to categorize healthy participants into left-lateralized (n = 264), right-lateralized (n = 31), and ambilateral (n = 31). Remarkably, they found no significant between-group differences in cognitive skills, measured via self-reports (number of fluently spoken languages, presence of university training, and presence of artistic activities) and tests (linguistic processing speed, verbal fluency, and general intelligence). Mellet et al. (2014) described that weak lateralization—that is, no clear hemispheric dominance in either the left or right hemisphere—was associated with worse performance in several cognitive components, including language skills. In left-handers, we found that the number of errors during the reading of word lists increased in relation to the degree of atypical lateralization for both language production (ρ = −.24 and −.23) and inhibitory control (ρ = .21 and .3) (Villar-Rodríguez, Cano-Melle, et al., 2024). In a set of left-handers different from that previous study, we also described a slightly better performance (η_p_^2^ = 0.14) among typically lateralized individuals compared with atypically lateralized ones—indifferently of them being right-lateralized or weakly lateralized—in cognitive tasks involving spatial processing, reading, articulation, and verbal reasoning (Cano-Melle et al., 2025). Similarly, research conducted in children has hinted at a link between atypical lateralization and poorer vocabulary and reading skills (Groen et al., 2012), as well as lower verbal IQ (Everts et al., 2009). Still, Lidzba et al. (2011) reported the opposite: that atypical rightward involvement during a comprehension task was associated with higher verbal IQ scores. In clinical populations, the presence of atypical language lateralization has been linked to certain language disorders, such as dyslexia (Bishop, 2013), although this association has not been consistently replicated (Wilson & Bishop, 2018).

Also relevant is the “crowding hypothesis,” which suggests that behavioral outcomes depend not on the isolated lateralization of functions but on their combined organization across hemispheres. In this regard, Gerrits et al. (2020) studied the combined lateralization phenotype of typically left functions (language production and praxis), and typically right functions (visuospatial attention, face recognition, and emotional prosody). Interestingly, they found no between-group differences in general cognitive performance—measured via the Repeatable Battery for the Assessment of Neuropsychological Status (RBANS)—when comparing typically (n = 39) and atypically (n = 24) lateralized for language production. However, individuals who presented two deviating functions from the full typical or full atypical segregation (n = 10) had significantly worse cognitive scores (η_p_^2^ = 0.22). In clinical populations, inconsistent lateralization of language components has been observed in individuals with developmental language impairments (Bradshaw et al., 2020).

In conclusion, although group-based statistics mostly support the notion of a unitary lateralization of language components, case-by-case data highlight the existence of individual exceptions. Also, recent studies have pointed at these hemispheric inconsistencies being relevant for cognitive performance. In the current study, we used fMRI to analyze the hemispheric lateralization of three different language components (production, comprehension, and reading) in three different cerebral regions (respectively, inferior frontal gyrus or Broca’s area, temporoparietal cortex or Wernicke’s area, and posterior fusiform gyrus or visual word form area). We chose tasks validated in Spanish because of possible cross-linguistic differences. Therefore, we used the verb generation and comprehension tasks developed by our group (Sanjuán, Bustamante, et al., 2010; Sanjuán, Forn, et al., 2010), as well as an adaptation of the overt reading task employed by Carreiras et al. (2007). It is worth noting that these tasks differ in sensory modality, with two being visual (verb generation and reading) and one auditory (comprehension). They also differ in their semantic demands: semantic processing is required in verb generation and comprehension, but to a lesser extent in reading (particularly in Spanish, given its transparent grapheme-to-phoneme correspondence). The examined Regions-of-Interest (ROIs) were the same as the ones in the original studies, except for the reading task, for which we used an external ROI (Chen et al., 2019) that overlaps with the two maxima reported by Carreiras et al. (2007) in the visual word form area (VWFA). Additionally, we conducted analyses at the inferior frontal gyrus for all three language components. This was done in a sample of non-right-handed individuals, preselected to be rich in examples of atypical language lateralization. This population has been reported to show a substantially high proportion of hemispheric inconsistencies (Labache et al., 2020), so we used this preselection in hopes of having enough inconsistently lateralized participants in our sample. Our two main objectives were (1) to describe the hemispheric consistency/inconsistency of the lateralized cerebral regions theoretically supporting these language components and (2) to assess the potential association between overt reading performance for words and pseudowords, and hemispheric consistency/inconsistency plus leftward/rightward dominance. We hypothesized that hemispheric inconsistencies between production–comprehension (Lee et al., 2008; Parker et al., 2022; Woodhead et al., 2021) and production–reading plus comprehension–reading (Gurunandan et al., 2020) would be found. That is, that inconsistencies of all possible types would exist. However, differences in particular proportions could shed some light into the specific drivers of hemispheric lateralization. Comprehension emerging as the most inconsistent component would support previously described two-factor models (Hickok & Poeppel, 2007; Parker et al., 2022). A higher inconsistency of reading, however, would align with evolutionary perspectives, hinting at differential neuroplasticity potentials (Gurunandan et al., 2020). We also hypothesized that hemispheric inconsistencies would be more frequent among atypical individuals (Labache et al., 2020), and that worse reading performance would be related to both rightward language dominance (Bishop, 2013; Cano-Melle et al., 2025; Everts et al., 2009; Groen et al., 2012; Villar-Rodríguez, Cano-Melle, et al., 2024) and hemispheric inconsistencies (Bradshaw et al., 2020; Gerrits et al., 2020; Vallortigara & Rogers, 2020).

## 2 Methods

### 2.1 Participants

Sixty-nine (69) participants were included in this study. All participants were left-handed (n = 59) or mixed-handed (n = 10) according to the Edinburgh Handedness Inventory/EHI (Oldfield, 1971). The EHI was scored following the recommendations by Bryden (1977), with scores categorized as follows: 10–23 (right-handed), 24–37 (mixed-handed), and 38–50 (left-handed). All participants were capable of writing only with their left hand. In our sample, EHI ranged from 30 to 50 (mean ± SD = 42.4 ± 4.7). When using the Laterality Quotient (LQ), an alternative scoring method for the EHI (Oldfield, 1971), LQ values in our sample ranged from −100 to 0 (mean ± SD = −74.2 ± 24.6), which corresponds to the left-handed range of LQ. We chose to include only non-right-handers in order to increase the variability of language lateralization in the sample, as this population has been demonstrated to have a notably higher incidence of strongly atypical (right) lateralization of language (Mazoyer et al., 2014; Pujol et al., 1999). However, it should be noted that the conclusions drawn from this particular population may not be generalizable to right-handed individuals, as previous studies have highlighted other lateralization differences between left- and right-handers apart from different incidences (Johnstone et al., 2021). Fluid intelligence was assessed using the WAIS-IV matrix reasoning subtest (Wechsler, 2012), with scores—scaled to age, with a range restriction of 1 to 19—ranging in our sample from 6 to 19 (mean ± SD = 12.1 ± 2.8). We selected this nonverbal intelligence test to control for its possible confounding effects on reading performance (Peng et al., 2019). Age ranged from 17 to 45 years old (mean ± SD = 22.8 ± 5.8). The sample included 30 males and 39 females.

None of the participants reported a history of head injury resulting in loss of consciousness, or any psychiatric or neurological disorders. Written informed consent was obtained from all participants for being included in the study, following a protocol approved by the Universitat Jaume I Ethics Committee (reference: CD/03/2021). All methods and procedures were conducted in accordance with the approved guidelines and current regulations, including the Declaration of Helsinki.

### 2.2 Cohort acquisition

Participants included in this study were selected based on an initial fMRI screening acquisition in which every willing healthy non-right-handed person over 16 years old was invited to participate, totaling 174 persons. In this first session, lateralization of language production was screened using real-time data (BrainWave software, GE HealthCare Technologies Inc.) from a verb generation task (see next section). The BrainWave software statistically models your predefined contrast of interest for the functional volumes as they are acquired—without any preprocessing—and continuously updates the corresponding *t*-maps in native space. Looking at these maps, the researcher visually judged whether the real-time activation in the inferior frontal gyrus (IFG) was potentially atypical; that is, whether it seemed to be right-lateralized or not clearly lateralized. If a participant was considered potentially atypical, they were invited to participate in a different fMRI session to take place in the future. Potentially typical participants were also invited to participate in this new fMRI session, until matching the number of potentially atypical who agreed to keep participating. Thus, this screening aimed to select approximately half of the sample as atypically lateralized for language production. As for the potentially typical participants, we prioritized inviting those whose age and sex roughly matched the potentially atypical participants. As a result of this screening, 45 potentially typical and 45 potentially atypical participants (25.86% of the 174 screened participants, which is in line with published incidences of atypical lateralization among left-handers), were invited to participate in the next fMRI session. Note that this categorization—which includes ambilateral cases that will be defined by LI cut-offs different from 0 in other studies—does not reflect the categorization used in the current study, which was based on a left/right lateralization split only.

Out of these 90 participants, only 69 were finally included in this study. The reasons for these 21 exclusions were many. First, the fMRI session included several sequences and tasks apart from those described in the present study, which resulted in time constraints and not being able to acquire all three language tasks in 15 participants. Second, technical malfunctions of the acquisition materials (response grip for comprehension and/or microphone for reading) led to not being able to confirm the task compliance of both tasks in five participants. Finally, one participant had to be discarded due to an incidental medical finding in their brain scan.

Regarding these 69 participants, 28 were also included in the following publications: Cano-Melle et al. (2025); Villar-Rodríguez, Cano-Melle, et al. (2024); Villar-Rodríguez, Davydova, et al. (2024); Villar-Rodríguez, Marin-Marin, Avila, et al. (2024); and Villar-Rodríguez, Marin-Marin, Baena-Pérez, et al. (2024). On a per study basis, 60 were included in Villar-Rodríguez, Marin-Marin, Baena-Pérez, et al. (2024), 54 were included in Villar-Rodríguez, Cano-Melle, et al. (2024), 63 were included in Villar-Rodríguez, Marin-Marin, Avila, et al. (2024), 54 were included in Villar-Rodríguez, Davydova, et al. (2024), and 43 were included in Cano-Melle et al. (2025).

### 2.3 Language paradigms

#### 2.3.1 Verb generation task

Expressive language function was assessed using an fMRI verb generation task (Sanjuán, Bustamante, et al., 2010). We selected this task for consistency with our first study on language lateralization in left-handers (Villar‐Rodríguez et al., 2020). Also, this task has been part of our in-house presurgical mapping protocol in tumoral and epilepsy patients for more than 15 years.

The task consists of a block design paradigm with active and control conditions. During the active condition, participants were presented with a series of nouns and asked to say the first verb that came to mind upon seeing each word. In the control condition, they were instructed to read aloud visually presented pairs of letters. The task was administered using E-prime 2.0 (https://pstnet.com/products/e-prime) and included six active and six control blocks. Each block lasted 30 seconds with each stimulus displayed for 1500 ms, followed by a blank inter-stimulus interval of 1500 ms. Before performing the task in the scanner, participants received detailed instructions and completed a 2-minute practice trial. Stimuli were presented via MRI-compatible googles (VisuaStim Digital, Resonance Technology Inc.) and verbal responses were played through a noise-cancelling microphone (FOMRI III +, Optoacoustics Ltd.) to ensure task compliance. The task was conducted in Spanish.

#### 2.3.2 Comprehension task

Comprehensive language function was assessed using an fMRI sentence verification or comprehension task developed by our team. Importantly, this task has demonstrated left-dominant activations in the temporoparietal areas of right-handed Spanish-speaking persons (Sanjuán, Forn, et al., 2010) and has been part of our in-house presurgical mapping protocol in tumoral and epilepsy patients for more than 15 years.

This task consists of a block design paradigm with active and control conditions. During the active condition, participants listened to short statements and were requested to determine whether they were true or false (e.g. “cats bark” or “strawberries are red”). In the control condition, they heard pairs of letters and indicated whether the letter “A” was present. The task was administered using E-prime 2.0 (https://pstnet.com/products/e-prime) and included 6 active and 6 control blocks (30 seconds each), with 6 statements per active block and 10 letter pairs per control block. Before performing the task in the scanner, participants received detailed instructions and completed a 2-minute practice trial. Stimuli were presented via an MRI-compatible headset (VisuaStim Digital, Resonance Technology Inc.) and manual responses were registered using an MRI-compatible response grip (ResponseGrips, NordicNeuroLab) to ensure task compliance. All participants responded using their left hand (index press = “true” or “A present”; thumb press = “false” or “A not present”). The task was conducted in Spanish.

#### 2.3.3 Reading task

Reading language function was assessed using an fMRI reading task, largely based on Carreiras et al. (2007). We selected this task primarily for two reasons. First, it has been shown to reliably elicit ventrotemporal/VWFA activation (Carreiras et al., 2007), which is relevant for studying lateralization shifts (Gerrits et al., 2019). Second, from a phonetic perspective, Spanish is considered a highly transparent language, meaning that spelling closely corresponds to sound. In contrast, English is an opaque language, where the relationship between graphemes and phonemes is inconsistent. This distinction might be relevant because phonetic transparency has been related to activation differences during fMRI reading tasks, although mainly when comparing non-alphabetic and alphabetic languages (Brignoni-Perez et al., 2020; Mei et al., 2013; for a review, see Cao, 2016)). The current task, which was originally designed and validated with Spanish native speakers, is consistent with the participants in our sample.

This task consists of a block design paradigm with active and control conditions. During the active condition, participants were presented with a series of words and pseudowords and were asked to read them aloud. In the control condition, they were shown strings of hash symbols (#) and instructed to say the word “casa” (Spanish for “house”) aloud for each string. The task was administered using E-prime 2.0 (https://pstnet.com/products/e-prime) and included six active and six control blocks. Each block lasted 30 seconds, with each stimulus displayed for 500 ms, followed by a blank inter-stimulus interval of 2500 ms. Active blocks included three words and three pseudowords. Words were selected from the EsPal database (Duchon et al., 2013) based on high imaginability and frequency. Pseudowords adhered to Spanish phonology and syllabic structure and were drawn from a previous study by our group (Palomar-García et al., 2023). Hash strings were matched in length to the words and pseudowords used in the active blocks. Before performing the task in the scanner, participants received detailed instructions and completed a 2-minute practice trial. Stimuli were presented via MRI-compatible goggles (VisuaStim Digital, Resonance Technology Inc.) and verbal responses were played through a noise-canceling microphone (FOMRI III +, Optoacoustics Ltd.) to ensure task compliance. The task was conducted in Spanish.

### 2.4 Reading measures

Reading proficiency was assessed outside the scanner using the word reading subtest of the PROLEC-SE-R battery (Cuetos Vega et al., 2016), which evaluates reading processes in Spanish. Participants were required to read (1) 4 lists of words, each containing 24 items classified as short familiar, long familiar words, short unfamiliar, and long unfamiliar words; and (2) 2 lists of pseudowords, each comprising 24 short and 24 long pseudowords. Responses were recorded using a microphone, capturing both accuracy (i.e., correct pronunciation on a per-word basis) and speed (i.e., time taken to read the full list) for each list. Notably, the acquired reading speed data exhibited a non-normal distribution (Shapiro–Wilk test of normality resulted in a *P* < .01 for all lists) with the presence of potential outliers (eight participants had an outlier slow reading speed in at least one of the lists, according to the 1.5 interquartile method). To address this, all speed data were log_10_-transformed.

### 2.5 Image acquisition

Images were acquired on a 3T General Electric Signa Architect magnetic resonance imaging scanner using a 24-channel head coil. All slices were acquired in strict sagittal plane. A 3D structural MRI was acquired for each participant using a T1-weighted magnetization-prepared rapid gradient-echo sequence (TR/TE = 8.5/3.3 ms; flip angle = 12º; matrix = 512 × 512 × 384; voxel size = 0.47 × 0.47 × 0.5 mm). For fMRI acquisition, gradient-echo T2*-weighted echo-planar imaging sequences were used to capture 144 functional volumes for the verb generation task (TR/TE = 2500/30 ms; flip angle = 70º; matrix = 64 × 64 × 30; voxel size = 3.75 × 3.75 × 4 mm), 144 functional volumes for the comprehension task (TR/TE = 2500/30 ms; flip angle = 70º; matrix = 64 × 64 × 30; voxel size = 3.75 × 3.75 × 4 mm), and 180 functional volumes for the reading task (TR/TE = 2000/30 ms; flip angle = 70º; matrix = 64 × 64 × 27; voxel size = 3.75 × 3.75 × 4.5 mm). Functional slices were acquired in interleaved sequence with strict axial orientation, aiming to full cortical coverage. Participants were instructed to minimize jaw movements during their responses to reduce motion artifacts during scanning. To further mitigate motion-related artifacts, the participants’ heads were stabilized within the coil using foam pads.

### 2.6 Image processing

The processing of functional images was performed using the Statistical Parametric Mapping software package (SPM12; Wellcome Trust Centre for Neuroimaging, London, UK) and MATLAB (version R2018b, MathWorks, Natick, MA). The default preprocessing pipeline was followed, including: (a) alignment of functional data to the AC‐PC plane using the anatomical image; (b) head motion correction, realigning and reslicing functional images to the mean functional image; (c) coregistration of the anatomical image to the mean functional image; (d) re‐segmentation of the anatomical image; (e) spatial normalization of functional images to MNI (Montreal Neurological Institute) space at a 3 mm^3^ resolution; and (f) spatial smoothing with a 4-mm full-width-at-half-maximum (FWHM) Gaussian kernel. Regarding head motion, out of 32,292 functional volumes, only 39 volumes presented a displacement in any direction higher than 1 mm or an angular motion higher than 1°, and only 2 volumes presented a displacement higher than 2 mm or an angular motion higher than 2°. No volume presented a displacement higher than 3 mm or an angular motion higher than 3°. General linear models (GLMs) were defined for each of the three functional tasks (verb generation, comprehension, and reading) by contrasting active > control blocks. The BOLD (Blood‐Oxygen‐Level‐Dependent) signal was estimated by convolving each task’s block onsets with the canonical hemodynamic response function (HRF). Six motion realignment parameters were included as nuisance regressors, and a high‐pass filter (128 seconds) was applied to contrast images to account for low-frequency drifts.

### 2.7 Individual functional lateralization and hemispheric consistency/inconsistency

Functional lateralization during each task was assessed using the Laterality index (LI), computed via the bootstrap method implemented in the LI-toolbox for SPM12 (Wilke & Lidzba, 2007). The LI quantifies the proportion of hemispheric activation differences for each individual using the formula: [(L − R)/(R + L)] × 100, where L represents the number of significantly active voxels in the left hemisphere mask, and R corresponds to the number in the right hemisphere mask. The LI ranges from + 100 (total left lateralization) to −100 (total right lateralization), providing a measure of both direction and degree of functional hemispheric dominance for a given task. The bootstrap method (Wilke & Schmithorst, 2006) calculates multiple iterations of LIs—up to 10,000—at different statistical thresholds, resulting in a weighted mean that takes said thresholds into account. This approach overcomes methodological concerns such as the arbitrary selection of statistical thresholds or the choice of voxel count vs. voxel intensity (Seghier, 2008). Bootstrap method was applied with the default toolbox settings (no optional clustering nor variance weighting).

**Fig. 1.**
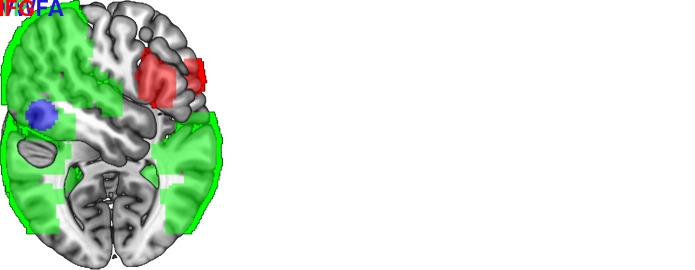
ROIs used for the LI calculations. IFG = inferior frontal gyrus, TP = temporoparietal, VWFA = visual word form area. Note that the VWFA and TP ROIs overlap, with an 84% of the VWFA ROI sharing voxels with the TP ROI.

We selected the LI ROIs based on previous lateralization and language research. As one of our objectives was to dichotomically categorize participants as either left- or right-dominant for each task, we focused on ROIs that yielded strong lateralization values. For the verb generation task, we examined the LI of the inferior frontal gyrus regions responsible for transforming the phonological information of speech into the specific motor commands needed for its articulation, specifically the pars opercularis and pars triangularis (Price, 2012), as defined by the maximum probability Harvard-Oxford atlas (Desikan et al., 2006; Frazier et al., 2005; Goldstein et al., 2007; Makris et al., 2006). This is the ROI we have used in prior studies assessing the lateralization of the verb generation task in Spanish speakers (Cano-Melle et al., 2025; Villar-Rodríguez, Cano-Melle, et al., 2024; Villar-Rodríguez, Davydova, et al., 2024) (bilateral count = 4,765 voxels). For the comprehension task, we assessed the temporoparietal region, including Brodmann areas 22, 37, 39, 40, 41, and 42, following our original description of the most strongly lateralized ROI on this task by Sanjuán, Forn, et al. (2010) in Spanish speakers (bilateral count = 38,119 voxels). For the reading task, we analyzed the VWFA using a 12-mm radius sphere centered at the MNI coordinates (−45, −57, −12) reported by Chen et al. (2019), a region that is robustly connected to the language network and critical for reading (bilateral count = 1,522 voxels). For additional comments and analyses regarding the validity of the VWFA ROI, check the Supplementary Material and Supplementary SFig. 1. Finally, due to the presence of inferior frontal activation in all tasks, we also calculated the LI of the inferior frontal gyrus for the comprehension and reading tasks. For a representation of all three ROIs, see Figure 1.

Participants were then categorized as hemispherically consistent (all three language components lateralized to the same hemisphere) or hemispherically inconsistent (one component lateralized in the opposite direction to the others). We used an LI threshold of 0 to determine hemispheric dominance. Therefore, a consistent individual had three positive or three negative LIs, while an inconsistent individual had two positive LIs and one negative LI, or vice versa. Additionally, we calculated LI distances between the three functions by computing the absolute difference in LI between (1) verb generation and comprehension, (2) verb generation and reading, and (3) comprehension and reading. The sum of these three LI distances provided an index of overall hemispheric consistency/inconsistency for every individual, with higher values indicating greater divergence among the three functions. So, for example, a participant presenting LIs of production = +60, comprehension = + 20, and reading = −30 would present a production–comprehension LI distance of 40, a production–reading LI distance of 90, and a comprehension–reading LI distance of 50. Thus, the total LI distance for that participant would be 40 + 90 + 50 = 180. Post hoc, complementary to this index, we also calculated the “distance to 0”. “Distance to 0” informs us about how strong is the overall lateralization of these three language components by computing the sum of its absolute LI values. Using the previous example, “distance to 0” would equal 60 + 20 + 30 = 110.

### 2.8 Statistical analyses

Voxel-wise whole-brain activations during the verb generation, comprehension, and reading tasks were analyzed. One-sample *t* tests (voxel-wise *P* < .05, uncorrected) were conducted for the “active > control” contrast, with participants grouped based on their lateralization index (LI > 0 for left-lateralized and LI < 0 for right-lateralized) for each task.

The association between the functional lateralization of the three tasks was assessed using Spearman’s correlations. Additionally, Spearman’s correlations were calculated between inferior frontal ROIs across all three tasks, as well as within tasks—during comprehension and reading—between inferior frontal ROIs and their relevant functional ROI. We also conducted an additional ANOVA to examine differences in the magnitude of lateralization between frontal and posterior regions, while also accounting for task. This was done by inputting the absolute values of LIs as an independent variable, while including ROI (frontal vs. posterior) and Task (comprehension vs. reading) as within-subject factors.

Significant differences in the proportion of consistency/inconsistency among left-dominant (three left-lateralized functions, or two left-lateralized and one right-lateralized) and right-dominant (three right-lateralized functions, or two right-lateralized and one left-lateralized) individuals were examined using a *χ*^2^ test. Furthermore, a chi-square goodness-of-fit test was performed to determine whether the diverging function among inconsistent individuals was equally distributed. Post hoc, we also computed (1) an ANOVA model to test possible associations between “distance to 0” (dependent variable) and consistency/inconsistency (factor 1) plus leftward/rightward dominance (factor 2) of language; and (2) a repeated-measures ANOVA model to test possible differences in “distance to 0” (dependent variable) between the three different language tasks (within-subject factor) among the consistent left-lateralized group.

Performance on the word and pseudoword reading lists (see “Reading measures” section) was analyzed using two repeated-measures ANOVA models (one for accuracy and one for speed). Each model included word familiarity (familiar/unfamiliar/pseudoword) and word length (short/long) as within-subject factors, and consistency/inconsistency as a between-subject factor. Age and fluid intelligence were included as covariates of no interest. We opted for a three-factor model because of the potential relevance of word length and familiarity effects on reading performance. These effects may indicate an over-reliance on sub-lexical processes during reading, which have been associated with reading difficulties and disorders (Borghesani et al., 2020; Provazza et al., 2019). In our previous study, we detected both effects in interaction with hemispheric lateralization (Villar-Rodríguez, Cano-Melle, et al., 2024). Therefore, similar interactions with hemispheric consistency/inconsistency were anticipated. Regarding the inclusion of age and fluid intelligence as covariates, it responds to the following. First, the age in our sample ranges from 17 to 45 years old. Given the decline of processing speed due to aging—and the potential impact of this on other cognitive domains such as reading (Salthouse, 1996)—we considered it appropriate to control for its effect in these analyses. Fluid intelligence also had a broad distribution in our sample (ranging from 6 to 19 scalar score, in an absolute range of 1 to 19). Fluid intelligence has been positively correlated with performance in a broad range of cognitive domains, including reading (Peng et al., 2019). Thus, we also found appropriate to control for its effect on these analyses. Notably, statistical significance of the presented results was unaffected by the inclusion/removal of these covariates.

Post hoc, the association between LI distances and reading time was tested using Spearman’s correlations. To further assess the role of hemispheric consistency vs. dominance in reading performance, two additional repeated-measures ANOVA models were conducted. These models were identical to the previous ones for accuracy and speed but included hemispheric dominance (left/right) as an additional between-subject factor.

## 3 Results

### 3.1 Associations between the LIs of language production, comprehension, and reading

**Fig. 2.**
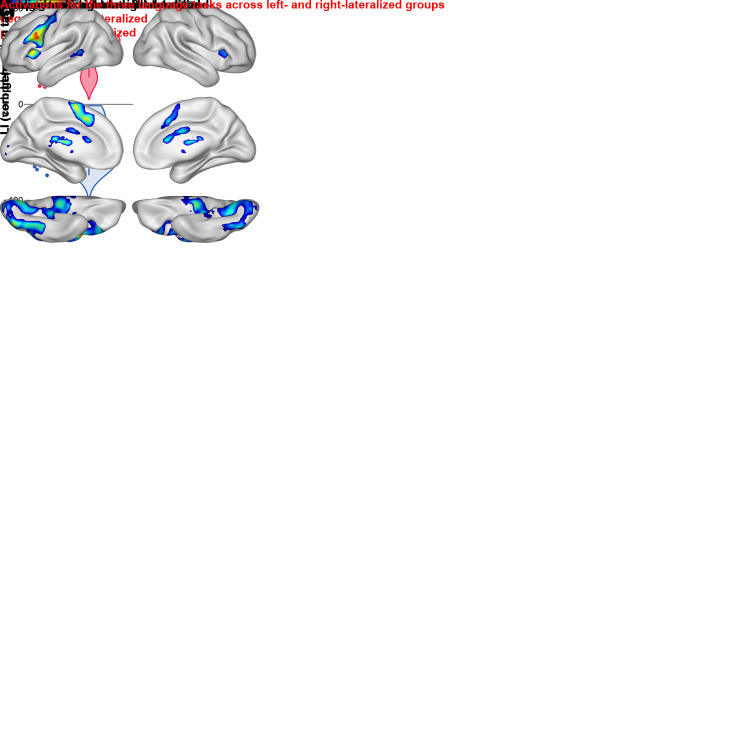
Verb generation, comprehension, and reading tasks. (a) Voxel-wise whole-brain one-sample *t* tests for the active > control contrast during the verb generation, comprehension, and reading tasks. Left-lateralized groups (LI > 0) are shown on the left, while right-lateralized groups (LI < 0) are shown on the right. Voxel-wise *P* < .05; uncorrected. Color bars represent *t* value, with the black line indicating the threshold for voxel-wise FWE-correction. If there is no black line, then no voxel survived the FWE correction. L = left hemisphere, R = right hemisphere. (b) Violin plots for the LI of the verb generation, comprehension, and reading tasks. Red plot = left-lateralized group, blue plot = right-lateralized group.

Group-wise cerebral activations during the verb generation, comprehension, and reading tasks are depicted in Figure 2a (one-sample *t* tests; voxel-wise *P* < .05, uncorrected), together with violin plots for the main LIs of the tasks (Fig. 2b). The verb generation task primarily activated the inferior frontal gyrus (both pars triangularis and opercularis), anterior insula, supplementary motor area, and middle temporal gyrus. The comprehension task showed extensive activations in the inferior frontal gyrus (extending into the left precentral gyrus), anterior insula, supplementary motor area, superior+middle+inferior temporal gyri, hippocampus, and lingual cortex (extending into the calcarine sulcus). The reading task activated a broad frontal network (including inferior frontal gyrus, precentral gyrus, postcentral gyrus, anterior insula, and supplementary motor area), together with the supramarginal gyrus, superior+middle temporal gyri, superior+inferior occipital gyri, and fusiform gyrus (corresponding approximately to the VWFA).

**Fig. 3.**
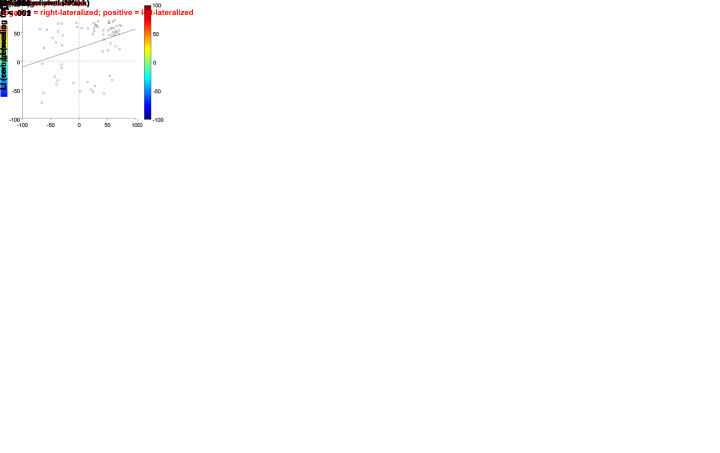
Correlations between the lateralization of verb generation, comprehension, and reading tasks. (a) Dispersion plots illustrating Spearman’s correlations between pairs of LIs from the language tasks, together with the heatmaps displaying the relationship with the third language LI via the color grid. Warmer colors (e.g., red) indicate leftward lateralization, while cooler colors (e.g., blue) indicate rightward lateralization. The color grid was generated using cubic interpolation to estimate values between the data points. (b) Dispersion plot and heatmap depicting correlations between LIs calculated in the inferior frontal gyrus for all three tasks. (c) Dispersion plots showing intra-task Spearman’s correlations in the comprehension task (between the temporal+parietal cortex and the inferior frontal gyrus) and in the reading task (between the VWFA and the inferior frontal gyrus).

**Fig. 4.**
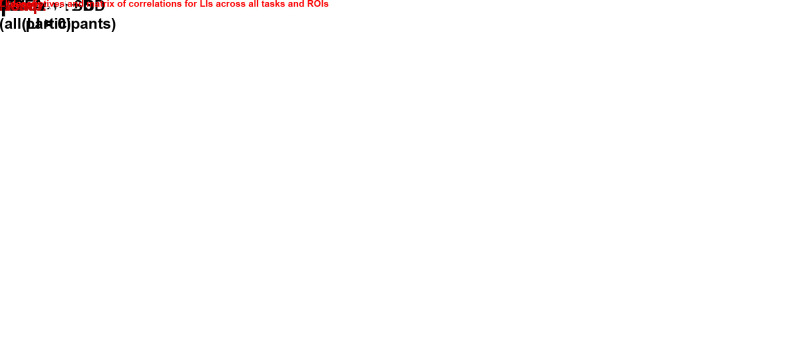
Descriptive statistics and correlation values for the LIs of the verb generation, comprehension, and reading tasks. The left matrix summarizes the means and standard deviations for all computed LIs, while the right matrix displays Spearman’s correlation coefficients between the LIs of all explored regions in their respective language tasks. All correlations were statistically significant at *P* < .05. SD = standard deviation, Prod. = production, Comp. = comprehension, Read. = reading, IFG = inferior frontal gyrus, TP = temporal and parietal cortex, and VWFA = visual word form area.

Hemispheric lateralization was assessed using Laterality Indexes (LIs) calculated for the inferior frontal cortex in the three tasks, for the temporoparietal region in the comprehension task, and for the VWFA in the reading task. Figure 3 includes two-variable dispersion plots plus three-variable heat maps for the different LIs (Fig. 3a+3b), and dispersion plots for the intra-task LIs (Fig. 3c). Figure 4 displays the descriptive statistics plus correlation values between the different LIs. First result is that all LIs were significantly correlated with each other, irrespective of task and ROI (see Fig. 4; for a list of all *P* values, refer to the repository in the data availability statement). LIs derived from the verb generation and comprehension tasks showed a strong correlation between them all. However, the correlations involving the reading task were considerably weaker, even when comparing the same ROI across tasks—inferior frontal gyrus—or the two intra-task reading ROIs. In fact, the dispersion plots suggest that the associations involving the reading task may be driven by data clustering in the typical quadrant (both functions left-lateralized), as data points appear evenly distributed across the other three quadrants/phenotypes, something that does not seem to be the case when plotting production and comprehension (see Fig. 3a, left graphs). In other words, correlations involving reading might be reflecting that most participants show congruent lateralization, rather than the tasks being truly correlated. A similar conclusion emerges from the heat maps (see Fig. 3a, second and third heat maps): production and comprehension LIs display a clear alignment: warmer colors (leftward lateralization) correspond to the leftward lateralization of the other function, while cooler colors (rightward lateralization) correspond to the rightward space of the graph. Reading LI (first heat map), however, shows no clear or consistent organizational pattern with respect to the lateralization of the other two functions. Thus, in our left-handed sample, only verb generation and comprehension exhibit a strong correlation in hemispheric lateralization, whereas reading lateralization is relatively independent of the other two functions—except in the most typically left-dominant participants.

The IFG lateralization in verb generation and comprehension was also strongly correlated, with data clustering particularly evident in the strongly lateralized quadrants, both leftward and rightward (see Fig. 3b, left graph). The IFG lateralization during reading was also significantly correlated with that of verb generation, albeit to a lesser extent. As illustrated in the heat map (Fig. 3b, right graph), the frontal component of reading shows a stronger correspondence with verb generation than the VWFA component observed previously. This is evident in the way warmer colors (leftward lateralization) predominantly cluster on the right, while cooler colors (rightward lateralization) are mostly on the left. Additionally, a strong intra-task correlation was found between frontal and temporoparietal LIs during comprehension, although this relationship seems less evident among weakly lateralized individuals (Fig. 3c, left graph). A moderate correlation was also found between frontal and VWFA LIs during reading, mainly driven by participants with left-lateralization in both regions (Fig. 3c, right graph). Interestingly, very few individuals exhibited rightward VWFA activation without concurrent rightward IFG activation, and even those who did had at most a slightly leftward IFG. In conclusion, IFG activation during comprehension and reading demonstrates a strong lateralization correspondence with IFG activation during production, and in the case of reading, this correspondence is even stronger than with the VWFA. However, in both tasks, correspondence is not perfect, and discrepancies still exist. The intra-task regional correspondence appears to be notably stronger in comprehension than in reading.

Finally, we conducted an additional ANOVA to examine differences in the magnitude of lateralization between frontal and posterior regions, while also accounting for task. Consistent with the means presented in Figure 4, a significant main effect of region was detected (*F*_1,68_ = 32.1; *P* < .001), indicating that the frontal ROI showed stronger lateralization (mean absolute LI = 57.9) than the posterior ROIs (mean absolute LI = 46.6), regardless of task.

### 3.2 Hemispheric consistency/inconsistency of language production, comprehension, and reading

For subsequent analyses, we focused on the following language components: the IFG during verb generation, the temporal+parietal cortex during comprehension, and the VWFA during reading. Assessing these components, we qualitatively defined hemispheric consistency/inconsistency. Participants were categorized as consistent if all three components were lateralized to the same hemisphere, or inconsistent if one component was lateralized differently from the others. This classification resulted in 44 consistent and 25 inconsistent individuals (see Table 1). Notably, inconsistency was significantly more frequent (*χ*^2^ = 19.07, *P* < .001) when a left-lateralized component diverged from two right-lateralized components (n = 17) than when the reverse occurred (n = 8). Among rightward-dominant individuals (three right-lateralized functions, or two right-lateralized and one left-lateralized), the incidence of inconsistency was 70.8%, whereas among leftward-dominant individuals (three left-lateralized functions, or two left-lateralized and one right-lateralized), it was only 17.8%. This indicates that inconsistency was strongly associated with an overall rightward-dominant language organization. Additionally, a chi-square goodness-of-fit test revealed that, among these rightward-dominant inconsistencies, reading was the most frequent diverging component, on the verge of statistical significance (*χ*^2^ = 5.765, *P* = .056). This tendency aligns with the previous observation that reading lateralization does not clearly fit the lateralization of production and comprehension.

**Table 1.** Distribution of consistent and inconsistent groups according to hemispheric lateralization during the verb generation, comprehension, and reading tasks.

	Hemispheric lateralization (mean ± standard deviation LI)		
Participants (n)	Production Inferior frontal gyrus	Comprehension Temporal and parietal cortex	Reading Visual word form area
Consistent (n = 44)			
37	L (60.5 ± 14.7)	L (50.2 ± 17.1)	L (52.3 ± 14.3)
**7**	**R** **(−44.1 ± 20.5)**	**R** **(−44.7 ± 21.1)**	**R** **(−36.1 ± 24.8)**
Inconsistent (n = 25)			
3	L (38 ± 13.9)	L (43.7 ± 15.5)	R (−44.7 ± 12)
2	L (44 ± 35.4)	R (−20 ± 24)	L (56 ± 5.7)
3	R (−16.4 ± 15.3)	L (46.3 ± 26.3)	L (60 ± 10.8)
**10**	**R** **(−45.2 ± 19)**	**R** **(−40.8 ± 18.9)**	**L** **(48 ± 16.4)**
**5**	**R** **(−45 ± 27.6)**	**L** **(23.8 ± 19.6)**	**R** **(−44 ± 12.1)**
**2**	**L** **(45 ± 17)**	**R** **(−37 ± 8.5)**	**R** **(−19.5 ± 10.6)**

Among the consistent and inconsistent groups, white rows correspond to leftward-dominant individuals (in whom at least two out of three components are left-lateralized), while rows in bold correspond to rightward-dominant individuals (in whom at least two out of three components are right-lateralized).

L = left, R = right.

We also explored the possibility that the degree of lateralization—quantified by the “distance to 0”—could be interacting with its consistency and direction. An ANOVA resulted in a significant interaction between hemispheric consistency/inconsistency and leftward/rightward dominance on the total “distance to 0” (*F*_3,65_ = 4.86; *P* = .031). Specifically, it showed that the pattern of language lateralization was generally weaker among inconsistent individuals—both leftward and rightward ones—and consistent rightward individuals, in comparison with consistent leftward individuals (see Table 2, column for Total). A very similar interaction was found for the distance to 0 of language production alone (*F*_3,65_ = 8.303; *P* = .005), whereas weaker lateralization during reading was associated only with rightward dominance (*F*_3,65_ = 7.553; *P* = .008). Finally, an additional ANOVA restricted to consistent left-dominant individuals confirmed that the distance to 0 for production was significantly higher in comparison with those for comprehension and reading (*F*_2,35_ = 6.369, *P* = .004; respective pair-wise tests: *P* = .003 and *P* = .02).

**Table 2.** Averaged total and task-wise “distance to 0”, grouped by hemispheric consistency/inconsistency and left/right language dominance.

	Distance to 0 (mean ± standard deviation)			
Participants (n)	Total	Production	Comprehension	Reading
Consistent (n = 44)	156.95 ± 34.14	57.87 ± 16.61	49.33 ± 17.67	49.74 ± 17.11
Left-dominant (n = 37)	163.03 ± 27.43	60.49 ± 14.68	50.22 ± 17.14	52.32 ± 14.29
Right-dominant (n = 7)	124.83 ± 49.06	44.06 ± 20.47	44.67 ± 21.14	36.1 ± 24.77
Inconsistent (n = 25)	123.76 ± 25.01	40.73 ± 21.25	36.43 ± 19.62	46.6 ± 15.73
Left-dominant (n = 8)	123.42 ± 16.58	31.41 ± 21.46	38.76 ± 21.99	53.25 ± 11.51
Right-dominant (n = 17)	123.92 ± 28.59	45.11 ± 20.3	35.33 ± 19.02	43.47 ± 16.75

### 3.3 Behavioral effects of hemispheric consistency/inconsistency on reading speed

**Fig. 5.**
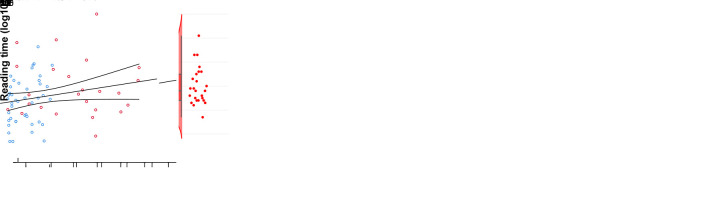
Behavioral effects of hemispheric consistency/inconsistency of language components (inferior frontal gyrus during production, temporal+parietal cortex during comprehension, and VWFA during reading) on reading speed of words and pseudowords lists. (a) Rain plot illustrating the effect of hemispheric consistency/inconsistency on reading time. Boxes represent the median ± inter-quartile range, while whiskers indicate the data range. (b) Dispersion plots depicting the Spearman’s correlation between reading speed and global LI distance (calculated across verb generation, comprehension, and reading tasks; on the left) and specific LI distance between verb generation and reading tasks. Reading times are represented as transformed values following a log_10_ conversion. Blue dots = consistent individuals; red dots = inconsistent individuals.

We investigated whether hemispheric consistency/inconsistency influenced behavioral performance during the reading of word lists that varied in word length and familiarity, including pseudowords. Two repeated-measures ANOVAs were performed: one for accuracy and one for speed. No significant effect was found for reading accuracy, with an averaged high accuracy in both the consistent (95.2 ± 2.6%) and inconsistent groups (95.4 ± 3.1%). However, a significant main effect of consistency/inconsistency on reading speed was observed (*F*_1,65_ = 4.78; *P* = .032; ηp² = .07), indicating that the consistent group read the lists faster than the inconsistent group, regardless of word familiarity or length (Fig. 5a). Moreover, a significant positive correlation existed between reading time and the LI distance of the three language components (*ρ*_67_ = .273; *P* = .023; see Fig. 5b left graph), where LI distance denotes the absolute difference between the LIs of each function (see Methods 2.7 for its calculation and an example). So, the greater the hemispheric divergence was, the slower the reading. To further describe this relationship, we computed the three individual LI distances that contributed to the global distance: verb generation and comprehension, verb generation and reading, and comprehension and reading. Interestingly, only the LI distance between verb generation and reading remained significantly correlated with reading speed (*ρ*_67_ = .269; *P* = .025; see Fig. 5b right graph). This suggests that reading speed (but not accuracy) is negatively affected by hemispheric inconsistency between the inferior frontal cortex, measured during language production, and the VWFA, measured during reading.

In a different study analyzing a subset of this left-handed sample (50 out of the 69 current participants were included in that study), our findings indicated that reading accuracy declined with atypical hemispheric dominance (Villar-Rodríguez, Cano-Melle, et al., 2024). Given the current observation of an association between hemispheric inconsistency and an atypical rightward dominance of language, we aimed to determine the extent to which both current and prior behavioral results could be attributed to hemispheric inconsistency versus rightward dominance. To address this, we replicated the previously presented ANOVAs while incorporating dominant hemisphere—that is, the predominating hemisphere across the three language components. See Table 3 for behavioral data grouped by both consistency/inconsistency and left/right dominance. In line with the findings in our prior study, the analysis of reading accuracy revealed a significant interaction between word familiarity, word length, and dominant hemisphere (*F*_2,62_ = 4.17; *P* = .02; ηp² = .12). Specifically, rightward-dominant individuals exhibited more frequent reading errors when reading long, unfamiliar words. When examining reading speed, the inclusion of dominant hemisphere did not alter the aforementioned main effect of consistency/inconsistency (*F*_1,63_ = 5.71; *P* = .02; ηp² = .08) and did not introduce any new effects. This suggests that while reading speed is negatively affected by hemispheric inconsistency between the inferior frontal cortex and VWFA, worse reading accuracy is more closely linked to an atypical hemispheric dominance of language.

**Table 3.** Accuracy and speed data for the reading of words and pseudowords lists, grouped by hemispheric consistency/inconsistency and left/right language dominance.

	Accuracy (mean ± standard deviation in %)					
Participants (n)	Short familiar words	Short unfamiliar words	Short pseudowords	Long familiar words	Long unfamiliar words	Long pseudowords
Consistent (n = 44)	99.15 ± 2.31	98.86 ± 2.6	94.22 ± 4.6	99.62 ± 1.21	96.31 ± 5.02	82.77 ± 7.76
Left-dominant (n = 37)	99.44 ± 1.45	99.21 ± 1.66	94.82 ± 3.98	99.89 ± 0.69	97.75 ± 3.2	82.54 ± 8.27
Right-dominant (n = 7)	97.62 ± 4.72	97.02 ± 5.22	91.07 ± 6.56	98.21 ± 2.23	88.69 ± 6.23	83.93 ± 4.45
Inconsistent (n = 25)	99 ± 1.82	98 ± 4.98	94.17 ± 3.99	99 ± 2.76	94.83 ± 7.54	87.67 ± 6.07
Left-dominant (n = 8)	98.96 ± 1.93	97.92 ± 4.45	93.75 ± 3.86	99.48 ± 1.47	96.35 ± 3.48	87.5 ± 3.15
Right-dominant (n = 17)	99.02 ± 1.82	98.04 ± 5.33	94.36 ± 4.15	98.77 ± 3.22	94.12 ± 8.85	87.74 ± 7.14
	Speed (mean ± standard deviation in seconds)					
Participants (n)	Short familiar words	Short unfamiliar words	Short pseudowords	Long familiar words	Long unfamiliar words	Long pseudowords
Consistent (n = 44)	13.14 ± 3.01	14.14 ± 4.38	15.48 ± 3.06	16.82 ± 4.81	17.45 ± 3.74	16.06 ± 3.85
Left-dominant (n = 37)	13.64 ± 2.96	13.89 ± 4.37	15.8 ± 3.07	16.65 ± 4.81	17.78 ± 3.82	16.66 ± 3.82
Right-dominant (n = 7)	10.50 ± 1.67	15.42 ± 4.6	13.75 ± 2.51	17.72 ± 5.1	15.68 ± 2.85	12.89 ± 2.27
Inconsistent (n = 25)	14.81 ± 4.39	14.15 ± 3.61	18.2 ± 5.27	16.46 ± 3.83	19.83 ± 5.6	18.3 ± 5.3
Left-dominant (n = 8)	14.06 ± 3.98	14.87 ± 4.57	16.95 ± 3.62	17.16 ± 4.94	18.45 ± 4.18	17.29 ± 3.98
Right-dominant (n = 17)	15.17 ± 4.64	13.81 ± 3.18	18.79 ± 5.89	16.12 ± 3.32	20.49 ± 6.17	18.78 ± 5.88

## 4 Discussion

In the present work, we examined the hemispheric consistency of language production, comprehension, and reading in a sample of 69 left-handed individuals, of whom 24 were rightward-dominant for language. From our results, we extracted six relevant conclusions: (1) lateralization was stronger in frontal regions than in posterior regions; (2) language production and comprehension present a strong correspondence in their LIs; (3) concordance of reading LIs with production and comprehension LIs was, at most, moderate; (4) hemispheric inconsistency was substantially more prevalent among right-dominant individuals (70.8%) than among left-dominant ones (17.8%); (5) both right-dominance and hemispheric inconsistency were linked to a weaker overall lateralization; and (6) inconsistent individuals exhibited slightly slower reading speeds, particularly when the inconsistency occurred between production and reading.

The first conclusion of this study is that among left-handed participants with consistently left-hemisphere language lateralization—that is, individuals with all three language functions left-lateralized—the LIs are greater in magnitude in the frontal lobe than in posterior regions. Moreso, when comparing intra-task ROIs, this phenomenon occurred regardless of hemispheric dominance. The pattern of higher LIs in frontal vs. posterior regions is consistent with the Hickok and Poeppel model, as well as with previous findings in both right- and left-handers (Hickok & Poeppel, 2007; Parker et al., 2022; Stroobant et al., 2009; Zaca et al., 2012). A different aspect is the co-lateralization of functions. At the group level, one of the most relevant results of this study is a clear and strong correlation between the functional lateralization of language production and comprehension. This was evident not only in their respective inferior frontal and temporoparietal regions, but also when considering only the inferior frontal region for both tasks. This finding was somewhat unexpected according to both the Hickok and Poeppel model, and the recent proposal by Parker et al. (2022) and Woodhead et al. (2021) of two different factors in language lateralization. However, it is consistent with previous studies, such as Häberling et al. (2016), which had already shown this strong co-lateralization using two semantic tasks, namely the word generation and synonym judgment tasks. In our study, the relevant stimuli of the comprehension task consist of spoken sentences, whereas those of the verb generation task are written words. Both share the requirement of semantic processing, at the word level (verb generation) and at the sentence level (comprehension). This common factor could explain the high correlation between them (and, incidentally, the lower correlation with a non-semantic task like reading words and pseudowords). The two-factor model by Parker et al. (2022) described common paths between the tasks and the factors, which resulted in the sentence decision task (the closest to our comprehension task) having loadings in both factors. Still, the strong correspondence between both LIs in our results partially supports the idea that semantic processing is a common factor for both regions.

Our findings also emphasize that reading appears to be the least hemispherically consistent component when examined alongside production and comprehension. While the latter two functions showed a clear correlation, their associations with reading—whether its lateralization was assessed in the VWFA or the IFG—were also significant but much weaker. In these associations, the statistical significance seemed to arise mainly from the fact that most participants exhibited both components in the left hemisphere, rather than from a true linear relationship between them. This diverges slightly from previous results by Gerrits et al. (2019) and Van der Haegen et al. (2012), which showed a stronger association between the LIs of the IFG during word generation and the VWFA during a lexical decision and a 1-back task. Still, this could be explained by the different characteristics of previous studies, such as the use of reading tasks that did not require overt reading, the higher proportion of participants with atypical language lateralization, or the preselection of participants based on an hemifield tachistoscopic task involving naming and reading—which could have led to an oversampling of individuals with congruent production and reading lateralization. Moreover, regarding their choice of a lexical decision task, a recent study has shown greater IFG involvement in this task compared with silent reading (Turker et al., 2025), which may increase co-lateralization. Therefore, these differences may account for the slightly lower co-lateralization observed in our reading task.

The exploration of our data on an individual basis also supported the notion of reading having a more inconsistent lateralization. A diverging reading component—where reading was left-lateralized in a rightward-dominant individual, or left-lateralized in a rightward-dominant individual—was the most frequent cause of hemispheric inconsistency, occurring in 52% of such cases. This can be understood under the wing of the results by Gurunandan et al. (2020), which point at reading having the highest neuroplastic potential among these three language components. This phenomenon may stem from how “recent” this function is from a phylogenetic perspective. The earliest forms of writing date back to approximately 5,000 years ago (Schmandt-Besserat, 2006), and reading only became widespread after the invention of the printing press (less than 600 years ago). Spoken language, by contrast, is believed to have been present for at least 135,000 years ago (Miyagawa et al., 2025). Therefore, it is extremely unlikely that strong genetic “boundaries” underlie the lateralization of reading, whereas such constraints are far more plausible for language production (Plaut & Behrmann, 2011). This discrepancy may also be attributed to differences in lateralization biases between occipital-focused perceptual functions and other cognitive functions. Karlsson et al. (2022) reported incongruency in 28% of cases between language production and face perception, and in 26% between production and body perception. These values are remarkably similar to the incongruency rates observed in our study between reading and production (26%) and between reading and comprehension (29%). Going back to our data, we also found a notable scarcity of individuals who exhibited a right-lateralized VWFA during reading without a concurrent right-lateralized IFG. That is, during reading, right-lateralized VWFA was accompanied most of the time by a right-lateralized IFG. This hints, in turn, at an ontogenetic explanation: reading is the latest language function to develop in the individual and, as such, it may be dependent on earlier development patterns (and thus orbit around a different lateralization factor). As to what are those earlier patterns, we can only speculate about the idea that the emergence of the VWFA may reflect the recycling of cortical territories that were already strongly connected with the IFG (Mahon & Caramazza, 2011). This pre-existing connectivity could have provided an efficient pathway for linking visual-orthographic processing with phonological and lexical representations (Bouhali et al., 2014). Taken altogether, our reading results could probably fit a two-factor model as proposed by Woodhead et al. (2021) and Parker et al. (2022), but not in the same structure as they suggest—which focuses on comprehension. Nonetheless, it should also be noted that these findings diverge from those of Labache et al. (2020), where reading was the most divergent component only in a subgroup of typical (leftward-dominant) individuals. In contrast, our data show that this effect is most evident among rightward-dominant individuals. We believe these discrepancies may be attributed to methodological differences between studies, such as the inclusion of right-handers (not present in our study), differences in the fMRI tasks used and their baseline conditions, and, most importantly, the different criteria to define language dominance groups (rightward vs. strongly atypical, and cutoff point vs. Gaussian modeling) or consistency/inconsistency (single ROI vs. multiple ROI).

The lack of correspondence between the different language tasks allows for distinguishing consistent individuals (those in whom the three language functions are controlled by the same hemisphere) from inconsistent individuals (those in whom one language function lateralizes to a different hemisphere than the other two), highlighting the potential need, under certain circumstances, for stronger interhemispheric transfer. We have found that hemispheric inconsistencies in language were significantly more frequent among rightward-dominant individuals. This replicates the findings by Labache et al. (2020) in a large sample of both left-handers and right-handers. Nevertheless, the incidences of inconsistencies among atypical vs. typical groups (70.8% and 17.8%) are notably higher than those found in their study for atypical vs. typical (40% and 4%; as discussed in the previous paragraph, we attribute this to methodological differences). Also, if we understand left-handedness as an approximate proxy for rightward dominance—given that left-handers present a higher incidence of rightward dominance than right-handers (Mazoyer et al., 2014; Pujol et al., 1999)—our findings are in line with those by Woodhead et al. (2021), who revealed that left-handers exhibit lower covariance between the lateralization of different language components compared with right-handers. Regarding the underlying mechanisms, we propose that differences in interhemispheric connectivity are a suitable explanation for this effect. With some exceptions (Westerhausen et al., 2025), atypical lateralization has been theorized to arise from hyperconnectivity through the corpus callosum, which could enable a more flexible hemispheric organization of language-related cognitive components (Labache et al., 2020; Tzourio-Mazoyer, 2016; Villar-Rodríguez, Marin-Marin, Baena-Pérez, et al., 2024; for an earlier proposal, see Gazzaniga, 2000). In fact, the weaker overall lateralization observed in the right-dominant and inconsistent groups is consistent with this proposed flexibility, and mirrors the pattern previously described in left-handers relative to right-handers (Johnstone et al., 2021). In support of this idea, recent studies confirm that atypically lateralized individuals present increased callosal volume and higher interhemispheric functional connectivity (Andrulyte et al., 2024; Labache et al., 2020; Villar-Rodríguez, Cano-Melle, et al., 2024; Villar-Rodríguez, Marin-Marin, Baena-Pérez, et al., 2024)—although others have failed to find a relationship between functional lateralization and callosal morphology (Westerhausen et al., 2025) or found it in a different direction (Josse et al., 2008; Karpychev et al., 2022; Westerhausen et al., 2006). Regarding the microstructure of the corpus callosum, the work of Karolis et al. (2019) and Zhu et al. (2024) highlights how these properties are closely linked to functional lateralization patterns. Thus, we can speculate that the significantly higher proportion of hemispheric inconsistencies among rightward vs. leftward lateralized individuals might be driven by the role of callosal connections and interhemispheric connectivity in shaping atypical hemispheric specialization.

Regarding the impact of hemispheric consistency/inconsistency on behavior, our data indicate that the inconsistent group presented a slightly slower reading speed than the consistent group, while showing no differences in reading accuracy. Moreover, LI distance, a rough measure of hemispheric inconsistency, was linearly associated with this small decrease in reading speed, particularly when considering the difference between the production and reading components. In other words, the greater the hemispheric inconsistency among language components, the slower the individual reads. This finding aligns with the idea that hemispheric specialization enhances processing speed and efficiency by minimizing the need for interhemispheric transmission, allowing cognitive functions to operate more seamlessly within the same hemisphere (Ringo et al., 1994; Rogers, 2000; Vallortigara & Rogers, 2020). Furthermore, our results are consistent with Bradshaw et al. (2020), who suggested that inconsistent lateralization of language components might increase the risk of developing language disorders. On top of that, we partially replicated our previous findings in Villar-Rodríguez, Cano-Melle, et al. (2024), which related atypical dominance of language to a slightly worse accuracy—but not slower speed—when reading long unfamiliar words. Thus, this part of our results also sums up to previous evidence suggesting that atypical language dominance may be associated with variability in language performance, or even the presence of language disorders (Everts et al., 2009; Groen et al., 2012; for a review, see Bishop, 2013). Taken altogether, our study supports the idea that variability in hemispheric organization within the language network—in terms of leftward vs. rightward dominance, or consistent vs. inconsistent—can translate to behavioral differences, affecting the efficacy (accuracy) or the efficiency (speed) of language processing. Still, we should be cautious about these conclusions. First, even if the behavioral differences were statistically significant, they were also small in effect size. And second, our sample consisted primarily of university students, a population with a relatively restricted cognitive and behavioral range, which may limit the generalizability of our findings to the broader population.

In conclusion, we showed that—among left-handers—the hemispheric lateralization of language is not unitary across its functional components. In our data, reading emerged as the most hemispherically diverging component, while production and comprehension had a very strong correspondence. We also linked atypical dominance of language with a higher likelihood of inconsistent language lateralization patterns, which in turn was related to worse reading accuracy (in the case of rightward language dominance) or slower reading speed (when the lateralization pattern was inconsistent).

## Supplementary Material

Supplementary Material

## Data Availability

The datasets generated and analyzed during the current study are available in the figshare repository, 10.6084/m9.figshare.27908685.v1.
