# Supplementary material for: Hemispheric consistency in language production, comprehension, and reading in typically and atypically lateralized left-handers: Implications for reading performance
Source: Imaging Neurosci (Camb). 2026 May 26;4:IMAG.a.1240. doi: 10.1162/IMAG.a.1240 (PMC13214573; doi:10.1162/IMAG.a.1240)
Supplement: Supplementary Material [file IMAG.a.1240_supp.pdf]

SUPPLEMENTARY MATERIAL

HEMISPHERIC CONSISTENCY IN LANGUAGE PRODUCTION, COMPREHENSION, AND READING IN TYPICALLY AND ATYPICALLY LATERALIZED LEFT-HANDERS: IMPLICATIONS FOR READING PERFORMANCE

1 Visual Word Form Area (VWFA) ROI

For the lateralization analyses of the reading task, we defined the VWFA using a 12-mm radius sphere centered at the MNI coordinates (−45, −57, −12) reported by Chen et al. (2019). However, previous research has shown that the location of the VWFA varies considerably across individuals (Glezer & Riesenhuber, 2013). This raises potential concerns about the validity of our ROI, mainly: (1) is the ROI successfully capturing peak activation within the ventral occipitotemporal cortex? and (2) is this also true for the right hemisphere, considering the original MNI coordinates are based on the left hemisphere?

To clarify this, we computed the activation maps (voxel-wise one-sample t tests at $P < .05$, uncorrected) for the left-lateralized group ($n = 52$) and the right-lateralized group ($n = 17$) (see SFig 1a). Note that both maps were limited in extension to the ventral occipitotemporal cortices (vOT) through a mask including the inferior temporal gyri, inferior occipital gyri, and fusiform gyri (see SFig 1b).

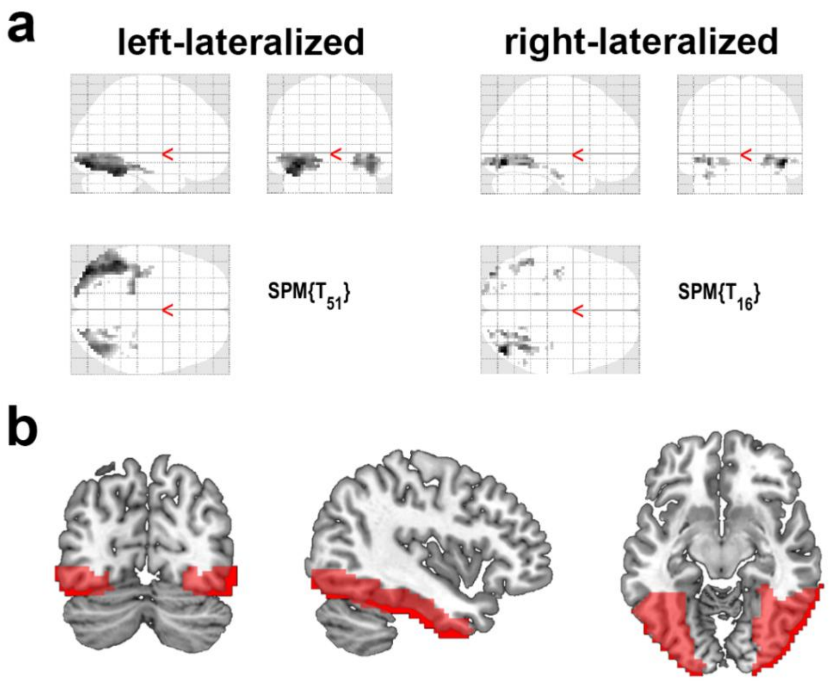

SFig. 1 VWFA and vOT. (**a**) glass-brain display of activation maps during the reading task for the left-lateralized group and right-lateralized groups, constrained to the vOT ($P < .05$, uncorrected). (b) vOT mask

Then, we considered the 5 local maxima (defined in MNI coordinates) in each hemisphere for each group activation map.

For the left-lateralized group:

- In the left hemisphere, the 3rd peak (−48, −52, −22) and the 5th peak (−45, −49, −19) were encompassed by the VWFA ROI
- In the right hemisphere, the 3rd peak (42, −64, −19) was encompassed by the VWFA ROI.

For the right-lateralized group:

- In the left hemisphere, the 2nd peak (−48, −52, −7) and the 3rd peak (−48, −46, −10) were encompassed by the VWFA ROI.
- In the right hemisphere, the 2nd peak (39, −55, −13) was encompassed by the VWFA ROI.

Therefore, although our VWFA ROI does not encompass all maxima, nor the absolute peak of each cluster, it successfully captured some of the local maximum activations.

For our next verification, we calculated a new Laterality Index (LI) during the reading task. For this alternate LI, we used the vOT ROI. This is a broader ROI than VWFA, so it should provide a wider representation of activations into the LI.

Surprisingly, VWFA and vOT LIs (respectively, mean ± SD = 29.9 ± 42, and 27.9 ± 38.5) were extremely similar, showing a very strong correlation ($r = .882$, $P < .001$). When using the vOT LI, our results involving correlations between the LIs of the different tasks and regions remained almost identical. On a case-by-case basis, using the vOT LI changed the lateralization from left to right or viceversa in only 5 participants (LI changes: from −26 to 31, −12 to 35, −5 to 34, 26 to −4, and 48 to −2). This, however, did not significantly affect subsequent results using these groupings:

- Among rightward-dominant participants, the incidence of inconsistency was still greater (72% with vOT, 70.8% with VWFA) when compared to leftward-dominant individuals (25.5% with vOT, 17.8% with VWFA).
- Reading was still the most frequent diverging component among right-dominant inconsistencies (it actually manages to reach statistical significance while using vOT; before, this effect had a $P = .056$).
- Degree of lateralization summed across the three tasks (“distance to 0”) also reached the same conclusions.
- Behavioral results regarding reading speed and accuracy showed the same trends: inconsistent individuals read slightly slower than consistent individuals

(P = .07 with vOT, P = .03 with VWFA), and right-dominant individuals made slightly more reading mistakes than left-dominant individuals (P = .002 with vOT, P = .02 with VWFA).

In conclusion, after conducting these checks, we consider it safe to affirm that our VWFA ROI appropriately represented the functional lateralization of the wider vOT. And importantly, the use of one ROI over the other does not substantially impact the results of the present study.

REFERENCES

Chen, L., Wassermann, D., Abrams, D. A., Kochalka, J., Gallardo-Diez, G., & Menon, V. (2019). The visual word form area (VWFA) is part of both language and attention circuitry. *Nature Communications 2019 10:1*, *10*(1), 1–12. https://doi.org/10.1038/s41467-019-13634-z

Glezer, L. S., & Riesenhuber, M. (2013). Individual Variability in Location Impacts Orthographic Selectivity in the “Visual Word Form Area.” *Journal of Neuroscience*, *33*(27), 11221–11226. https://doi.org/10.1523/JNEUROSCI.5002-12.2013
